# Bone Mass and Strength in Older Men With Type 2 Diabetes: The Osteoporotic Fractures in Men Study

**DOI:** 10.1359/jbmr.090725

**Published:** 2009-07-13

**Authors:** Moira A Petit, Misti L Paudel, Brent C Taylor, Julie M Hughes, Elsa S Strotmeyer, Ann V Schwartz, Jane A Cauley, Joseph M Zmuda, Andrew R Hoffman, Kristine E Ensrud

**Affiliations:** 1 School of Kinesiology, Laboratory for Musculoskeletal Health, University of Minnesota Minneapolis, MN, USA; 2 Division of Epidemiology, University of Minnesota Minneapolis, MN, USA; 3 Center for Chronic Disease Outcomes Research, Veterans Affairs Medical Center Minneapolis, MN, USA; 4 Department of Medicine, University of Minnesota Minneapolis, MN, USA; 5 Division of Epidemiology, University of Pittsburgh Pittsburgh, PA, USA; 6 Department of Epidemiology and Biostatistics, University of California San Francisco, CA, USA; 7 VA Palo Alto Health Care System and Division of Endocrinology, Stanford University Palo Alto, CA, USA

**Keywords:** bone geometry, osteoporosis, peripheral quantitative computed tomography, diabetes mellitus

## Abstract

The effects of type 2 diabetes mellitus (T2DM) on bone volumetric density, bone geometry, and estimates of bone strength are not well established. We used peripheral quantitative computed tomography (pQCT) to compare tibial and radial bone volumetric density (vBMD, mg/cm^3^), total (ToA, mm^2^) and cortical (CoA, mm^2^) bone area and estimates of bone compressive and bending strength in a subset (*n* = 1171) of men (≥65 years of age) who participated in the multisite Osteoporotic Fractures in Men (MrOS) study. Analysis of covariance–adjusted bone data for clinic site, age, and limb length (model 1) and further adjusted for body weight (model 2) were used to compare data between participants with (*n* = 190) and without (*n* = 981) T2DM. At both the distal tibia and radius, patients with T2DM had greater bone vBMD (+2% to +4%, model 1, *p* < .05) and a smaller bone area (ToA −1% to −4%, model 2, *p* < .05). The higher vBMD compensated for lower bone area, resulting in no differences in estimated compressive bone strength at the distal trabecular bone regions. At the mostly cortical bone midshaft sites of the radius and tibia, men with T2DM had lower ToA (−1% to −3%, *p* < .05), resulting in lower bone bending strength at both sites after adjusting for body weight (−2% to −5%, *p* < .05) despite the lack of difference in cortical vBMD at these sites. These data demonstrate that older men with T2DM have bone strength that is low relative to body weight at the cortical-rich midshaft of the radius despite no difference in cortical vBMD. © 2010 American Society for Bone and Mineral Research

## Introduction

Observational cohort studies have found that type 2 diabetes mellitus (T2DM) is associated with a 50% to 80% increased risk of hip fracture, as well as a 30% to 70% increased risk of fracture of the proximal humerus and foot.(1–3) Although there is awareness of the higher fracture rates among diabetic adults,(4,5) there are few data available on the factors responsible for this increased risk. Identifying these factors is a critical step in the development of potential interventions to prevent fractures among the growing segment(6) of the adult population with T2DM.

Most, but not all, cross-sectional studies have found average or even somewhat higher areal bone mineral density (aBMD, g/cm^2^) assessed by dual x-ray absorptiometry (DXA) in patients with T2DM compared with healthy controls, even after accounting for larger body size among diabetics.(7–9) These results are somewhat surprising given the increased fracture risk associated with T2DM. However, DXA studies that use aBMD as an outcome have several limitations. Measurement of aBMD essentially assumes that bone is an amorphous solid, for which size, shape, and the distribution of bone material within are irrelevant. For example, DXA-measured bone density does not account for bone dimensional changes or allow for separation of the cortical and trabecular bone compartments. This may be important in adults with T2DM for two reasons: (1) trabecular bone, which is disproportionately affected by T2DM,(10) may not be detected by DXA, and (2) bone *strength* can be reduced even when there are no changes (or even an increase) in *a*BMD because of geometric changes.(11) Other factors may influence bone strength in diabetics, including changes in bone material properties (i.e., enzymatic and nonenzymatic cross-links(12)), but bone material properties per se cannot be measured noninvasively.(13)

A 3D imaging technique, peripheral quantitative computed tomography (pQCT), has allowed for assessment of volumetric density of both cortical and trabecular compartments, as well as structural estimates of bone strength derived from cross-sectional geometry.(14,15) This technology has the potential to better classify skeletal properties. Few clinical studies have examined bone properties other than aBMD in patients with T2DM. Human studies exploring the association between T2DM and bone parameters other than aBMD have focused on spine bone volumetric BMD(16,17) but have not reported bone geometric parameters or estimates of cortical and trabecular bone strength. However, animal studies suggest that bone properties are compromised in rats with T2DM,(10,18) highlighting the importance of assessing these properties in humans.

The primary objective of this study was to examine the association between T2DM and bone volumetric density, geometry, and estimates of bone strength in community-dwelling older men. We hypothesized that men with T2DM would have lower bone strength relative to their body weight, particularly in highly trabecular regions such as the distal radius and distal tibia.

## Methods

### Participants

From March 2000 through April 2002, 5995 men who were at least 65 years of age were enrolled in the baseline examination of the prospective Osteoporotic Fractures in Men (MrOS) study.(19) Men were recruited from population-based listings in six areas of the United States: Birmingham, Alabama, Minneapolis, Minnesota, Palo Alto, California, the Monongahela Valley near Pittsburgh, Pennsylvania, Portland, Oregon, and San Diego, California.(19,20) Men with a history of bilateral hip replacement and men who were unable to walk without the assistance of another person were excluded. The institutional review boards of each center approved the study protocol, and written consent was obtained from all participants.

Men who returned for their second exam an average of 4.7 ± 0.4 years later were invited to participate in an ancillary study involving pQCT. Of the 1550 men who attended the second exam at the Pittsburgh and Minneapolis study sites, 1171 (76%) completed the clinic visit and agreed to participate in the pQCT ancillary study and are included in this analysis. This ancillary study was approved by the institutional review boards at the Minneapolis and Pittsburgh sites. All participants in the ancillary study signed a written informed consent for the pQCT portion of the study.

### Peripheral quantitative computed tomography measurements

Peripheral quantitative computed tomography (pQCT) was used to obtain slices (2.3 ± 0.2 mm) at the 4% and 66% sites of the left tibia and at 4% and 33% of the nondominant forearm (radius). Slices are taken as a percentage of limb length from the distal end of the relevant bone. The XCT 2000 device (Stratec, Inc., Pforzheim, Germany) and the XCT-3000 device (Stratec, Inc., Pforzheim, Germany) were used to obtain the scans in Pittsburgh and Minneapolis, respectively. The only difference between the 2000 and 3000 scanners is the gantry size. The same acquisition and analysis software was used to analyze scans at both sites. We performed a precision study using a European forearm phantom scanned three times at each site at 200, 100, and 50 mg/cc, respectively. Values on the two instruments were similar and within less tha 0.5% for total area at all mg/cc values and from 0.5% to 1.0% for total density.

Voxel size was 0.5 mm, and the scan speed was 25 mm/s. The anatomic reference line (distal edge of the tibial plafond) was determined by acquisition of a 30 mm planar scout view of the joint line. Data were analyzed according to the manufacturer's specifications. At the trabecular 4% sites, Contour mode 2 (169 mg/cm^3^) and Peel mode 1 (45% area) were used. Distal sites were assessed for total bone cross-sectional area (ToA, mm^2^) and total density (ToD, mg/mm^3^). Bone strength index (BSI, mg/mm^4^) was calculated as (ToA × ToD^2^)/1,000,000 as an index of bone compressive strength. At the more cortical 33% radius and 66% tibia sites, we used Contour mode 2 (169 mg/cm^3^) to determine whole bone properties and Cortmode 1 (710 mg/cm^3^) for cortical bone properties. A threshold of 280 mg/cm^3^ was used to determine the polar strength strain index (SSIp). At these cortical sites, we assessed total bone cross-sectional area (ToA, mm^2^), cortical area (CoA, mm^2^), and cortical density (CoD, mg/mm^3^). Polar strength strain index (SSIp, mm^3^) and section modulus (mm^3^) were calculated as estimates of bone bending strength,(15) and muscle cross-sectional area (MCSA, mm^2^) was measured (at the 66% tibia only). SSIp is a “density-weighted” section modulus value, whereas section modulus includes only geometric properties. An anthropomorphic phantom was scanned daily for quality assurance at both sites.

### Dual energy x-ray absorptiometry

Total body, lumbar spine (L1 to L4), and total femur aBMD and body composition (total body lean mass and total body fat mass) were measured at the second exam using a fan-beam dual-energy X-ray absorptiometry (QDR 4500 W, Hologic, Inc., Bedford, MA, USA). Standardized procedures for participant positioning and scan analysis were executed for all scans. All DXA operators were centrally certified on the basis of an evaluation of scanning and analysis techniques. Cross-calibration studies performed prior to the baseline MrOS visit found no linear differences across scanners, and the maximum percentage difference in mean total spine BMD between scanners was 1.4%.(21) Longitudinal quality control using daily scan data for standardized phantoms at each site indicated no shifts or drifts in scanner performance.

### Health history, lifestyle, and demographic data

Information on demographics, medical and family history, and lifestyle were obtained by questionnaire and interview by trained clinical staff at each site. Information regarding age and race (white/nonwhite) was collected.

Presence or absence of T2DM was assessed by a self-report of physician diagnosis of diabetes or current use (at exam 2) of diabetes prescription medications, including hypoglycemics, sulfonylureas, or insulin. Fasting glucose and A1C assays were not available at exam 2.

Weight was measured in indoor clothing without shoes using a calibrated balance beam or digital scale. Height was measured using a Harpenden stadiometer (DyFed, UK), and body mass index was calculated (kg/m^2^). Prior to pQCT measurements, tibia and forearm length were measured to the nearest millimeter with an anthropometric tape measure. Tibia length was measured from the tibial plateau to the medial malleolus, and forearm length was measured from the ulnar styloid process to the olecranon process. The mean of two measurements for each variable was used for the analysis.

Tests of physical performance are included as descriptive variables. Gait speed was determined as usual time to complete a 6 m course and expressed in meters per second. Time to complete five chair stands (seconds) and ability to stand from a chair without using arms (yes/no) also were recorded. Grip strength was measured twice by a handheld dynamometer (Jamar) in both the left and right arms.(22) The average grip strength in kilograms was used in this analysis.

For lifestyle factors, men were classified into current, past, or never smokers using data collected from their current and baseline exams. Physical activity was measured by computing the Physical Activity Summary Scale for the Elderly (PASE).(23)

### Statistical analyses

Differences in characteristics according to T2DM status were compared using analysis of variance for normally distributed continuous data and chi-square tests for categorical data.

Bone measures were expressed as continuous variables using linear regression models, and the least-squared means procedure was used to estimate the mean (95% confidence interval) for each bone parameter by T2DM status (yes/no). Data are presented for two models first adjusted for age groups (65 to 69, 70 to 74, 75 to 79, 80+), race and tibia/radial length (model 1), and an additional model with further adjustment for body weight (model 2). Additional models were run substituting either lean mass or fat mass or body mass index instead of body weight, and results were similar (data not shown). Therefore, only results from models 1 and 2 are presented.

## Results

### Baseline descriptive characteristics

A total of 1171 participants had pQCT scans and complete visit data at either the Minnesota (*n* = 540) or Pittsburgh (*n* = 631) MrOs sites, 16% (*n* = 190) of whom had T2DM. A majority of men in both groups were Caucasian (98%), with 1% of participants being African American and 1% multiracial/other. Men with and without T2DM were similar in age, height, and tibia and radius length (Table 1). As expected, those with T2DM were heavier (+6.2 kg, *p* < .001) and had a significantly higher average BMI (+2.3 kg/m^2^, *p* < .001). Diabetic participants also had higher absolute levels of total body lean and fat mass (see Table 1) and a slightly higher percent of body weight from fat mass (28.7 ± 5.4 vs. 27.0 ± 5.2, *p* < .001). Men with diabetes also were significantly less active, had a lower grip strength, and took longer to complete the chair stand (see Table 1). Slightly more than half the men with T2DM were taking statins compared with slightly less than half the men without T2DM. As reported previously for the whole MrOS cohort,(24) men with diabetes had significantly higher aBMD measured by DXA at all measured sites (+4% to +8%, *p* < .05 for all sites).

**Table 1:** Descriptive and Functional Characteristics of 1171 Older Men by Diabetes Status

	Type 2 diabetes	No diabetes	*p*
*N*	190	981	
Age (years)	76.9 ± 4.8	77.3 ± 5.2	.394
Caucasian (%)	98	98	.802
Height (cm)	173 ± 6.4	173 ± 7.0	.392
Weight (kg)	89.1 ± 14.9	82.9 ± 13.0	<.001
Body mass index (kg/m^2^)	29.9 ± 4.5	27.6 ± 3.8	<.001
Total body lean mass (kg)	60.8 ± 7.8	57.8 ± 7.4	<.001
Total body fat mass (kg)	25.6 ± 8.0	22.4 ± 6.8	<.001
PASE score	124.7 ± 65.1	144.7 ± 65.6	<.001
Maximum grip strength (kg)	38.6 ± 7.8	41.0 ± 8.0	<.001
Time to complete five chair stands (s)	12.3 ± 3.5	11.4 ± 3.4	.002
Unable to complete chair stands without using hands (%)	20%	7%	<.001
Statins (%)	56%	43%	<.001
Tibia length (mm)	400.2 ± 27.3	402.9 ± 25.7	.210
Radius length (mm)	284.2 ± 18.5	283.8 ± 16.1	.790

Values are mean ± SD unless otherwise noted.

### pQCT bone outcomes

#### Tibia

Tibial bone density, geometry, and strength estimates from model 1 (adjusting for age, race, clinic site, and tibial length) and model 2 (adding body weight to the model) are presented in Table 2. At the highly trabecular distal tibia (4% site), diabetic participants tended to have a smaller total bone area (−1% to −3%) but higher bone tissue density (+1% to +3%). Differences were significant for density before weight adjustments (*p* = .025) and for total area after adjusting for weight (*p* = .007). The slightly greater density in diabetics compensated for lower bone area, so there was no difference in estimates of compressive bone strength (BSI) between groups at this site after adjusting for body weight (*p* = .591, model 2; see Table 2).

**Table 2:** Tibial Bone Volumetric Density, Geometry, and Strength in Older Men by Diabetes Status

	Model 1 (adjusted for age, race, clinic site, and tibia length)		Model 2 (adjusted for age, race, clinic site, tibia length, and body weight)	
		
	Type 2 diabetes	No diabetes	Type 2 diabetes	No diabetes
4% Tibia				
Total area (mm2)a	1273 (1249–1297)	1284 (1274–1295)	1252 (1228–1276)	1288 (1278–1298)*
Total density (mg/cm3)^a^	305 (298–312)	297 (294–300)*	303 (296–310)	297 (294–300)
Trabecular density (mg/cm^3^)^a^	237 (231–243)	229 (227–232)*	235 (229–241)	230 (227–232)
BSI^a^^b^	120 (115–125)	114 (112–116)*	116 (111–121)	115 (113–117)
66% Tibia				
Total area (mm^2^)^a^	763 (749–776)	768 (762–773)	753 (740–767)	769 (764–775)*
Cortical area (mm^2^)^a^	338 (330–346)	334 (330–337)	330 (322–337)	336 (332–339)
Cortical density (mg/cm^3^)^a^	1060 (1055–1065)	1064 (1061–1066)	1060 (1054–1065)	1064 (1061–1066)
pSSI (mm^3^)^a^	3397 (3319–3476)	3377 (3343–3411)	3323 (3247–3399)	3391 (3359–3424)
Section modulus (mm^3^)^b^	3386 (3304–3468)	3375 (3340–3411)	3298 (3219–3377)	3392 (3358–3426)*
Muscle CSA^c^	7723 (7566–7879)	7455 (7387–7524)*	7486 (7347–7626)	7502 (7442–7562)

Values are mean (95% confidence intervals).

BSI = (total area × total density^2^)/1,000,000.

Significantly different from diabetes group; *P < .05; **P < .001.

a *N* = 1135.

b *N* = 1132.

c *N* = 1124.

Participants with diabetes also tended to have smaller total bone area (−1% to −2%) at the cortical 66% site of the tibia, which was significantly different only after adjusting for body weight (*p* = .031). Cortical area was similar between groups both before and after adjusting for body weight. Cortical bone density was not different between groups at this site in either model. The smaller total bone area translated to a lower bone bending strength (section modulus, −2.8%, *p* = .033) after adjusting for body weight. SSIp also tended to be lower in men with diabetes after adjusting for body weight (−2.5%) but was not significantly different (*p* = .106). Prior to adjusting for body weight, however, there was no difference in section modulus (*p* = .819) or SSIp between groups (*p* = .636). Diabetic participants also had significantly larger muscle CSA (model 1, *p* = .002), although the muscle size was appropriate for their higher body weight (model 2, *p* = .841).

#### Radius

Bone parameters at the radius followed a similar pattern as those at the tibia (Table 3). At the distal trabecular site, diabetics again tended to have a smaller bone area (−3% to −4%) and higher bone density (+3% to +4%), but there was no difference in compressive bone strength after adjusting for weight (*p* = .284). Differences in volumetric density were significant both before (*p* = .012) and after (*p* = .029) adjusting for body weight, whereas total area differences reached significance only after adjusting for body weight (*p* = .003). At the cortical 33% site, bone strength again was significantly lower relative to body weight (SSIp, −5.2%, *p* < .001; section modulus, −4.5%, *p* = .002) in men with T2DM because total (−3.4%, *p* = .002) and cortical (−2.8%, *p* = .019) bone areas were significantly smaller after adjusting for body weight. Cortical bone density was not different between groups in either model. Again, bone strength was not different prior to adjusting for body weight (see Table 3, model 1).

**Table 3:** Radial Bone Volumetric Density, Geometry, and Strength in Older Men by Diabetes Status (95% Confidence intervals)

	Model 1 (adjusted for age, race, clinic site, and radius length)		Model 2 (adjusted for age, race, clinic site, radius length, and body weight)	
		
	Type 2 diabetes	No diabetes	Type 2 diabetes	No diabetes
**4% Radius**				
Total area (mm^2^)	379 (368–389)	390 (385–394)	374 (363–384)	391 (386–395)*
Total density (mg/cm^3^)	364 (354–373)	350 (346–354)*	362 (352–372)	350 (346–355)*
Trabecular density (mg/cm^3^)	205 (198–211)	195 (193–198)*	203 (197–210)	196 (193–199)*
BSI*	50 (48–53)	48 (47–49)*	49 (47–52)	48 (47–49)
**33% Radius**				
Total area (mm^2^)	143 (140–146)	146 (144–147)	141 (138–144)	146 (145–147)*
Cortical area (mm^2^)	104 (102–107)	105 (104–106)	103 (101–105)	106 (105–107)*
Cortical density (mg/cm^3^)	1158 (1153–1163)	1160 (1158–1162)	1160 (1155–1165)	1159 (1157–1162)
Strength strain index (mm^3^)	355 (345–365)	364 (360–369)	349 (339–358)	365 (361–370)*
Section modulus (mm^3^)	348 (338–358)	356 (351–360)	341 (332–350)	357 (353–361)*

Values are mean.

BSI = index of compressive bone strength [(total area × total density^2^)/1,000,000],

Significantly different from diabetes group; *p < .05; **p ≤ 0.001.

*N* = 1126 for models at 4% radius; *N* = 1122 for models at 33% radius.

## Discussion

We examined bone volumetric density, geometry, and estimates of bone strength at cortical and trabecular sites of peripheral bones in older men by T2DM status. Contrary to our hypothesis, we found that estimated compressive bone strength was not lower in the trabecular regions of men with T2DM. However, bone strength was compromised in more cortical regions relative to body weight in diabetics. In trabecular regions, a higher bone tissue density compensated for the lower bone area in diabetics, resulting in a lack of difference in estimates of compressive bone strength at these sites. In contrast, the smaller bone area (i.e., smaller periosteal circumference) among men with T2DM translated to lower bone bending strength (relative to body weight) in cortical regions of the tibia and radius.

### What are the implications of the differences in bone strength?

Epidemiologic studies suggest that patients with T2DM are at increased risk of hip, foot, and ankle fractures despite having consistently higher bone mineral density measured by DXA.(3,9,25) The higher aBMD persists even after adjusting for body weight in most studies. As a result, the focus of fracture risk in T2DM is often on increased risk of falls. Indeed, patients with diabetes may have an increased risk of falls owing to complications from comorbidities associated with T2DM. For example, impaired vision and peripheral neuropathy are common complications of T2DM—which may compromise vision and proprioception, respectively, and subsequently increase risk of falls.(26,27) In our population, older men with T2DM showed reduced muscular strength and neuromuscular function, which also might increase the risk of falls.

Nevertheless, the risk of fracture is a function of *both* the load on the bone during the fall and the strength of bone itself.(28) Our data demonstrate that estimates of bone strength at cortical sites are low relative to body weight in patients with T2DM. In previous studies demonstrating higher aBMD in type 2 diabetics, the higher DXA aBMD values might be explained by the smaller bone area in men with diabetes—that is, an equivalent amount of bone material within a smaller bone area would show up as a higher aBMD when measured by DXA.(17,29) However, because bone bending strength increases as bone is distributed further from the neutral axis, this smaller bone area (and higher aBMD) can translate to a lower bone bending strength.(29) Although indices of bone bending bone strength (section modulus and SSIp) among men with T2DM in our population were normal in an absolute sense (i.e., prior to adjusting for body weight), the section modulus was low for body weight, which might increase the risk of fracture in the case of a fall.

Other insults to the mechanical competence of bone, not measurable noninvasively in humans, have been demonstrated in T2DM. For instance, Saito and colleagues(12) demonstrated age-related changes in enzymatic and nonenzymatic cross-links of bone collagen in the spontaneously type 2 diabetic rat without a decrease in aBMD. This study suggested that alterations in collagen properties may make bone of type 2 diabetics more susceptible to fracture and, paired with our findings, demonstrates the importance of studying both bone strength and falls risk in T2DM.

### What could cause the differences in bone density and geometry in diabetics?

Several factors could influence bone properties in diabetic patients, including altered mechanical load, adipose-derived hormones, hyperglycemia, and/or pharmaceutical treatment,(30) as well as other diabetic complications such as renal failure,(31) microvascular complications, and peripheral neuropathy,(32) Consistent with the general type 2 diabetic population, a majority of the men in our population with T2DM were overweight (45% BMI > 25) or obese (44% BMI > 30). There is some confusion in the literature as to the effects of excessive body weight on bone mass and strength. A high body weight generally is considered to be protective of bone mechanical competence largely based on DXA data that consistently show high aBMD in overweight and obese adults.(33) As reported in nondiabetic overweight children and adults,(34–36) the older men with T2DM in our study had high absolute bone strength—but that strength was low once adjusted for their higher body weight despite similar cortical bone volumetric density.

Excess body weight affects bone not only via mechanical pathways but also through secretion of hormones from adipose tissue. These adipose-derived hormones (e.g., leptin, adiponectin, and resistin) have conflicting effects on bone metabolism.(30) For example, serum leptin levels have been positively correlated and serum adiponectin levels have been negatively correlated with aBMD of the hip, lumbar spine, and distal radius,(37,38) whereas resistin may stimulate osteoclastogenesis.(39) Although we did not measure adipose-derived hormones in this study, it is possible that serum elevations in these hormones would partially explain the smaller periosteal diameter (represented by total bone area) at the midshaft of the tibia and radius in type 2 diabetics in this study. However, it is unclear why these hormones would preferentially impact the periosteal surface. In contrast, mechanical loading has been demonstrated to add bone to the periosteal surface at the midshaft of long bones.(40) Therefore, the larger bone diameter in nondiabetics could reflect the higher physical activity level. While these theories remain be tested in this population, it is unclear why our findings are in contrast with animal data showing *increased* periosteal diameter in a type 2 diabetic rat model.(10,18)

At the distal trabecular regions of the tibia and radius, bone density was significantly higher in patients with diabetes. We had hypothesized that trabecular bone density would be compromised owing to data showing that, in animals, T2DM primarily impacts trabecular bone.(10,18) Owing to the increased body weight, we speculated that diabetics would have a wider bone area at the distal site and thus require a lower density to maintain mechanical competence. In contrast, older men in our population with diabetes had an *increased* bone density and smaller bone area at the trabecular sites. Data from Krakauer and colleagues(7) may help to explain these findings. These authors demonstrated low bone turnover in six participants with T2DM from histologic data from transiliac bone biopsies.(7) A lower bone turnover in this population may help to explain the higher volumetric density at the highly trabecular distal sites in diabetics. Our findings are also congruent with bone histomorphometric studies of diabetic animal models that have found significantly impaired bone formation and decreased mineralizing surface and mineral apposition rate in diabetic rats compared with healthy controls.(41) ^(Liu, 2007, #3460)^ Low bone turnover, coupled with impaired bone formation, may explain our findings of low bone area and greater vBMD in this population. However, pQCT has limited resolution and is unable to assess trabecular thickness or connectivity. Studies using higher-resolution instruments are needed to further explore these findings.

It is also possible that hormonal, nutritional, or pharmaceutical factors or other diabetic complications (e.g., renal failure) that we did not measure may have influenced our findings. For instance, the majority of our participants were on some form of diabetes medication. While metformin may have a positive effect on bone,(42) adverse effects of a group of diabetic drugs (i.e., thiazolidinediones) also has been demonstrated in humans.(43,44) In addition, renal failure is associated with altered bone properties(45) and is a common complication of T2DM. Our data were not powered to adequately explore the effects of different medications, but future research should incorporate anti-diabetic drug use and other mediating factors when interpreting the effects of T2DM on bone strength.

### Strengths and limitations

There are several strengths to our study, including the unique focus on older men. Although it is estimated that one in four men will experience an osteoporotic fracture in their lifetime, few studies have focused on risk factors for osteoporosis and fracture in older men. Another strength of the current study was the assessment of volumetric BMD, bone geometry, and structural strength estimates that allowed for characterization of the underpinnings of bone strength differences in older men with and without T2DM. There are, however, several factors we could not address adequately that may have influenced our results. In particular, the duration or severity of diabetes were not assessed. We did not have information on the disease duration and were unable to adequately assess disease severity owing to a lack of hemoglobin A1C outcomes at exam 2. In our sample, there were no clear differences between men taking insulin versus other hypoglycemics (data not shown). However, we had a relatively small sample size in each strata, with only 27 men taking insulin. In addition, low testosterone and growth hormone levels are associated with T2DM and may influence bone stiffness.(46) As with any cross-sectional study, our data show associations only and cannot prove causation. Future prospective studies should explore the role of disease duration and severity on bone outcomes and include assessment of hormonal factors. Importantly, a majority of the sample were white men, so we are not able to generalize results to other ethnic populations. Finally, a potential source of bias is the self-report diagnosis of diabetes. However, a majority of participants who self-identified as having T2DM also were on relevant medication.

## Summary and Conclusions

While past studies have demonstrated greater aBMD in T2DM, the current study, using pQCT, showed that type 2 diabetics had low bone strength for body weight at predominantly cortical sites. These findings, combined with the propensity to fall, may help to explain the increased risk of fracture in patients with type 2 diabetes.
